# Comparative Analysis of Meat Quality and Flavor Among Four Categories of Mongolian Horses

**DOI:** 10.3390/foods15112044

**Published:** 2026-06-05

**Authors:** Yu Liu, Xuejiao Wang, Shuqi Gong, Manglai Dugarjaviina, Xinzhuang Zhang

**Affiliations:** 1 Equus Research Center of Inner Mongolia Agricultural University, College of Animal Science, Inner Mongolia Agricultural University, Nei Mongol Autonomous Region, Hohhot 010018, China; liuyu97@nmgnydxwxy.wecom.work (Y.L.); 19821171727@163.com (S.G.); dmanglai@163.com (M.D.); 2 Rural Revitalization Research Institute, Inner Mongolia Agricultural University, Nei Mongol Autonomous Region, Hohhot 010018, China; wangxuejiao881019@163.com

**Keywords:** Wushen Horses, Baicha Horses, Barhu Horses, Ujimqin Horses, meat quality, lipidomics, electronic tongue, electronic nose

## Abstract

This study aims to conduct a comparative analysis of the quality and flavor of meat from four categories of Mongolian horses (Wushen, Baicha, Barhu, and Ujimqin). Physicochemical indicators, electronic nose, electronic tongue, and lipidomics were used to characterize meat quality and flavor and to screen for differential markers. Results showed that Wushen Horses had the highest pH_45min_, serine, glutamic acid, total free amino acids (∑FAA), total non-essential amino acids (∑NEAA), total amino acids (∑TAA), NEAA/TAA, W2S sensor response, umami and richness values, and had the lowest cooking loss, EAA/TAA, EAA/NEAA, sourness, bitterness and aftertaste B values (*p* < 0.01). In contrast, Barhu Horses had the highest b*_45min_, C20:2 and saltiness values, and had the lowest W5S, W1S and W2W sensor responses (*p* < 0.01). Lipidomics identified 163 differential lipids (DELs) as potential markers, including LPC (18:2/0:0) and PC (16:0_16:0). Kyoto Encyclopedia of Genes and Genomes (KEGG) enrichment analysis showed DELs were significantly enriched in glycerolipid, linoleic acid, arachidonic acid and α-linolenic acid metabolism pathways. Correlation analysis indicated 23 DELs (e.g., carnitine C20:4) correlated positively with umami, W2S and richness, but negatively with shear force and cooking loss. In summary, our data show that among the four categories of Mongolian horses, Wushen Horses exhibited the best meat quality and flavor, while Barhu Horses showed the poorest. The differences in meat quality and flavor were closely associated with changes in lipid composition. This study provides direct molecular evidence from lipids for the variation in meat quality among Mongolian horses.

## 1. Introduction

Horse meat is a high-quality meat that has gained worldwide popularity due to its excellent nutritional value, unique flavor, and functional properties [1]. According to FAOSTAT [2], global horse meat consumption reached 810,960 tons in 2023, an increase of 17.90% from 2013. The development of the meat industry depends on the supply of high-quality meat, and texture and flavor are critical factors influencing consumer choice. Compared with other meats, horse meat is characterized by high protein, high iron, high unsaturated fatty acids, low fat, and low cholesterol [3]. It is also rich in α-linolenic acid, which is essential for human health, and has demonstrated benefits in preventing anemia, arteriosclerosis, and hypertension [4]. In addition, horse meat contains high levels of glycogen and glutamic acid, which impart sweet and umami tastes and contribute to its unique flavor [5]. Horse meat has a firm texture, a mild and non-greasy aroma, and is easier to digest than beef or mutton. The quality attributes of horse meat, including nutritional composition, flavor compounds, and texture [6], are key indicators for its evaluation. As consumer demand for high-quality meat increases, the assessment and improvement of horse meat quality and flavor have become research focuses.

China has abundant horse breed resources, with 29 local breeds. Seven of these, including the Debao pony and the Mongolian horse, are listed in the national protected livestock genetic resources inventory [7]. In 2025, China’s horse population reached approximately 3.67 million, ranking sixth globally. The horse industry chain is valued at about 70 billion RMB, representing an important part of China’s livestock sector. Notably, China is the world’s largest exporter of horse meat, with products sold to Japan, Kazakhstan, Russia, and other countries [8]. Mongolian horses are among China’s finest equine genetic resources, known for their strong adaptability, tolerance to rough feeding, easy fattening, and good endurance. Within this breed, four local populations—Wushen, Baicha, Barhu, and Ujimqin—have developed distinct genetic and phenotypic traits through long-term natural selection and regional adaptation. The Wushen horse, from Uxin Banner, is small but strong, suitable for riding and pack transport. The Baicha horse, from Hexigten Banner, has small, hard hooves and excels on mountain trails. The Barhu horse, from Hulun Buir, performs well in racing and lassoing and is a typical Mongolian horse. The Ujimqin horse, from the Xilingol grassland, has a large frame and is known for its superior coat color and stamina. Due to different geographical distributions and grassland types, meat quality may vary among these four groups [9,10]. However, research on horse meat quality is still limited. There is a lack of scientific data, standardized feeding practices, and an integrated industry chain. Studies specifically comparing meat quality among the four Mongolian horse populations are extremely rare. Understanding these quality differences is crucial for enhancing the market competitiveness of Mongolian horse meat and guiding consumer choices.

In recent years, advanced technologies such as electronic nose, electronic tongue, and lipidomics have provided powerful tools for food quality research. The electronic nose enables rapid and accurate detection of volatile flavor compounds, while the electronic tongue is used to analyze taste characteristics. Lipidomics allows comprehensive and systematic analysis of lipid composition and metabolic pathways in biological samples, offering robust tools for elucidating the molecular mechanisms underlying food quality. With this technology, the composition, content, and metabolic variations in lipids among different meat breeds can be thoroughly investigated, providing a scientific basis for meat quality evaluation and improvement [11]. Lin et al. [12] integrated targeted lipidomics and electronic nose to compare lipid composition and odor dynamics in chicken breasts with different fat levels during cold storage, demonstrating that OPLS-DA and correlation analysis effectively distinguished aroma differences. Li et al. [13] used electronic nose, electronic tongue, and lipidomics to study the effects of reheating on braised pork, identifying key differential lipids and volatile compounds via OPLS-DA and VIP analysis. These results indicate that the combination of electronic nose, electronic tongue, and lipidomics, together with OPLS-DA and VIP analyses, is well suited for meat flavor research and can compare flavor and lipid differences among different treatment groups. However, this combined approach has not yet been applied to analyze the quality and flavor characteristics of meat from the four types of Mongolian horses.

Currently, little is known about the meat quality characteristics of these four categories of Mongolian horses. To fill this knowledge gap, the present study analyzed the meat quality traits of the four categories and used lipidomics to identify differential lipids (DELs), with the aim of screening key candidate lipids associated with meat quality. Using established scientific methods, we provide an objective and precise evaluation of meat quality attributes and nutritional characteristics for each category. This study aims to lay a foundation for the utilization of Mongolian horse meat resources and product development, to support the conservation of local breed diversity, and to promote the development of the specialty horse meat industry.

## 2. Materials and Methods

### 2.1. Animals

The four categories of Mongolian horses selected for this study were the Wushen Horses, Baicha Horses, Barghu Horses and Ujumqin Horses, originating from Uxin Banner in Ordos, Nei Mongol Autonomous Region, China; Hexigten Banner in Chifeng, Nei Mongol Autonomous Region, China; New Barag Right Banner in Hulunbuir, Nei Mongol Autonomous Region, China; and East Ujimqin Banner in Xilingol League, Nei Mongol Autonomous Region, China, respectively. The grassland in Uxin Banner is a typical temperate desert steppe, dominated by plants of the families Compositae and Gramineae, with common species including *Allium mongolicum*, *Caragana korshinskii*, and *Arrhenatherum elatius*. The grassland in Hexigten Banner is a temperate steppe, dominated by plants of the families Compositae and Gramineae, with common species including *Agropyron cristatum*, *Leymus chinensis*, and *Medicago sativa*. New Barag Right Banner is located deep within the renowned Hulunbuir Grassland. It represents a typical temperate meadow steppe, dominated by plants of the families Gramineae, Leguminosae, Compositae, and Liliaceae, with common species including *Stipa* spp., *Leymus chinensis*, and *Agropyron cristatum*. East Ujimqin Banner is situated in the core area of the Xilingol Grassland. Its grassland type is primarily temperate meadow steppe, with common plants including *Leymus chinensis* and *Stipa baicalensis* [14].

### 2.2. Experimental Design and Sample Collection

The material for this study comprised longissimus thoracis (LT) muscle samples from 40 healthy castrated male horses, aged 11 months and with similar body weights (300 ± 50 kg). The horses were obtained from individual farmers in four regions: Uxin Banner, Ordos City; Keshiketeng Banner, Chifeng City; New Barag West County, Hulun Buir City; and East Ujimqin County, Xilingol League, Inner Mongolia Autonomous Region, China, with ten animals from each region. All horses had a pre-slaughter body weight of 300 ± 50 kg. From birth until 6–8 months of age, the foals suckled their mothers; thereafter, they were grazed on natural pastures together with their mothers and remained in good health. Animal management complied with Directive 2010/63/EU (2010) [15,16]. Prior to slaughter, under welfare conditions, the horses were transported by road over a short distance (maximum 4 h) to a commercial abattoir in East Ujimqin County, Xilingol League (Jixiang Grassland Food Co., Ltd., Xilingol League, China). During transport, the animals were kept in individual compartments to prevent injury. After arrival, they were held in separate pens in lairage for approximately 24 h for acclimatization. Following animal welfare procedures, the horses were fasted for 24 h, then stunned by electrical stunning at the abattoir and exsanguinated. Slaughter and carcass dressing were performed according to the specifications of European legislation (Council Regulation 1099/2009) [15,16]. A longissimus thoracis (LT) sample (12th–13th thoracic vertebra level) of about 2 kg was collected from each horse. At 45 min post-mortem, pH and meat color were measured on site. The LT samples were then vacuum-packed and stored at 4 °C for meat quality analysis, while another portion was preserved in liquid nitrogen for lipidomic analysis. After fully freeze-drying the samples of LT using a lyophilizer (Lyolab, Coolvacuum, Granollers, Spain), the contents of fatty acids, amino acids, and minerals were determined. The remaining LT samples were stored at −80 °C for further use. All experimental protocols were approved by the Animal Care and Use Committee of Inner Mongolia Agricultural University (No. NND2024009).

### 2.3. Meat Quality Analysis

Following Ivanković et al. [4], pH at 45 min post-mortem (pH-Testo205, Delihua, Shenzhen, China; 2 cm insertion in LT) and meat color at 45 min post-mortem (lightness, L*; redness, a*; yellowness, b*; 4 °C, CR-400 colorimeter, Konica Minolta, Tokyo, Japan) were measured immediately at the abattoir. The pH meter was calibrated at room temperature using standard buffers (pH 4.00, 6.86, and 9.18). Drip loss was determined by suspending 10 g LT samples at 4 °C for 12 h, blotting surface moisture, and reweighing; the value was expressed as the percentage of weight loss during storage. Cooking loss was evaluated by cooking 120 g LT samples to an internal temperature of 70 °C, cooling, blotting, and reweighing; the value was expressed as the percentage of weight loss during cooking. Shear force was determined using a C-LM3B tenderness meter (Northeast Agricultural University, Shenyang, China) on three strips prepared parallel to the muscle fiber direction, with the mean value reported. Water-holding capacity (WHC) was analyzed using an RH-1000 instrument (Guangzhou Runhu Instrument Co., Ltd., Guangzhou, China) with the filter-paper method. Texture profile analysis (TPA) parameters including cohesiveness, springiness, chewiness, and gumminess were measured on 1 cm-thick LT samples using a TMS-Touch texture analyzer (Food Technology Corporation, Sterling, VA, USA) according to Stanisławczyk et al. [16]. All determinations were performed in triplicate.

### 2.4. Determination of Chemical and Mineral Composition

The moisture content was assayed via direct drying (GB 5009.3-2016) [17]: 5 g LT samples were oven-dried (BPG-9420A, Shanghai Yiheng, Shanghai, China) at 105 °C to constant weight, with moisture percentage calculated from weight loss. Crude fat (3 g) was quantified through Soxhlet extraction using anhydrous diethyl ether as solvent, following GB/T 5009.6-2016 [18]. Ash content determination (GB/T 5009.4-2016) [19] which involved incinerating 5 g LT samples in porcelain crucibles at 550 °C in a muffle furnace (MFLC-16/12C, Tianjin Taisite, Tianjin, China) until constant weight. Protein content (1 g) was measured by Kjeldahl method with a KT260 analyzer (FOSS, Hillerød, Denmark), complying with GB 5009.5-2016 [20]. For mineral analysis, 5 g LT samples were pretreated following Seong et al. [3]. The samples were destroyed by dry ashing in a muffle furnace (MFLC-16/12C, Tianjin Taisite) for 12 h with a final temperature of 600 °C. The ash content was dissolved in 10 mL of 37% HCl and the distilled water (1:1 *v*/*v*) solution and was then filtered through filter paper. Then calcium (422.7 nm), phosphorus (470 nm), iron (248.3 nm), zinc (213.9 nm), copper (324.8 nm) and selenium (196.0 nm) were detected via ICP-OES (ZEEnit700P, Analytik Jena, Jena, Germany), with calibration curves established for each element.

### 2.5. Fatty Acids Profile

After freeze-drying, LT samples were ground into a homogeneous powder. Fatty acid methyl esters (FAMEs) were prepared according to the Chinese National Food Safety Standard GB 5009.168-2016 [21] and Ivanković et al. [4]. Briefly, approximately 0.3 g of freeze-dried LT powder was used for fatty acid extraction and methylation. Separation and quantification of fatty acids were performed using a gas chromatography-mass spectrometry system (Trace 1310-ISQ 7000, Thermo Fisher Scientific, Waltham, MA, USA) equipped with a TG-FAME capillary column (50 m × 0.25 mm × 0.20 μm). Individual fatty acids were identified by comparison with a 51-component FAME mix standard (NU-CHEK-PREP, Elysian, MN, USA). Individual fatty acids were identified and quantified based on their retention times and peak areas, with results expressed as relative percentages (%). The relative contributions of saturated fatty acids (SFA), monounsaturated fatty acids (MUFA), polyunsaturated fatty acids (PUFA), n-3 polyunsaturated fatty acids (n-3 PUFA), and n-6 polyunsaturated fatty acids (n-6 PUFA) were also calculated.

### 2.6. Amino Acid Composition

Following freeze-drying to a constant weight using a lyophilizer (Scientz-50ND, Scientz Biotechnology Co., Ltd., Ningbo, China), LT samples were analyzed for amino acid composition. The separation and detection of amino acids were performed using the method described by Pan et al. [22] with an amino acid analyzer (S-433D, SYKAM, Munich, Germany). Chromatographic separation was conducted at a constant flow rate of 1.7 mL/min. The elution program commenced with an initial 1 min isocratic hold of 100% solution A (0.2 mol/L sodium citrate, pH 3.0), followed by a 48 min linear gradient transition from 0% to 100% solution B (0.2 mol/L sodium borate, pH 9.8).

### 2.7. Electronic Nose and Electronic Tongue Analysis

The samples (5 g) were placed into 20 mL headspace sample vials after pretreatment according to the method described by Zhou et al. [23], and then incubated at 25 °C for 30 min to reach equilibrium before determination with a PEN3 portable electronic nose (NOVA Enose3, Beijing Innovate Technology Development Co., Ltd., Beijing, China). The machine parameters were set as follows: self-cleaning time of 60 s, injection flow rate was of 400 mL/min, and detection time of 200 s. The sensor signal values of 193–195 s were selected for data analysis when a steady status could be maintained. The electronic nose contains 10 metal oxide single thick film sensors, namely W1C (sensitive to aromatic, benzene), W5S (sensitive to nitrogen oxides), W3C (sensitive to ammonia and aromatic compounds), W6S (sensitive to hydrogen), W5C (sensitive to alkanes, aromatic compounds, and fewer polar compounds), W1S (sensitive to methane and hydrocarbons), W1W (sensitive to many terpenes and sulfide compounds), W2S (sensitive to alcohols, aldehydes, and ketones), W2W (sensitive to organic sulfides, aromatic compounds), and W3S (sensitive to long-chain alkanes). The results were reported as the average of three replicates.

Pretreatment was performed following the methods described by Bayinbate et al. [24]. The samples (30 g) were mixed with 150 mL of distilled water and centrifuged at 2265× *g* at 4 °C for 10 min, the water phase obtained was used for electronic tongue analysis. The taste characteristics of LT samples were analyzed with a C-SA402B taste sensor, E-tongue instrument (Intelligent Sensor Technology, Inc., Atsugi, Japan) that is equipped with five chemical sensors: C00 (bitterness and aftertaste bitterness), AE1 (astringency and aftertaste astringency), AAE (umami and richness), CT0 (saltiness), and CA0 (sourness), along with two reference electrodes.

### 2.8. Lipidomic Analysis

Lipidomics was conducted by Metware Biotechnology (Wuhan, China). LT samples were thawed on ice, homogenized with steel balls and 1 mL methanol/MTBE/internal standard solution, vortexed for 15 min, mixed with 200 μL water, and centrifuged (12,000× *g*, 4 °C, 10 min). A 300 μL supernatant was concentrated, redissolved in 200 μL solution, stored at −80 °C, and analyzed via LC-MS/MS.

Analyses were performed on an ExionLC AD UPLC coupled with a QTRAP^®^ 6500 + MS, using a Thermo Accucore™ C30 column. Mobile phases A (acetonitrile/water, 60:40, 0.1% formic acid, 10 mmol/L ammonium formate) and B (acetonitrile/isopropanol, 10:90, same additives) were run with a gradient program (0–20 min, 80:20 to 80:20 *v*/*v*) at 0.35 mL/min, 45 °C, 2 μL injection volume. ESI source parameters included 500 °C, ± 4500/5500 V voltage, 45/55/35 psi gas settings. QQQ/LIT scans and MRM transitions were optimized with Analyst 1.6.3 software.

Lipid identification was performed based on the MetWare in-house lipid database (MWDB) combined with the multiple reaction monitoring (MRM) mode of the QTRAP^®^ 6500 + MS system. The qualitative identification of lipids was achieved by matching the retention time (RT), precursor-product ion pair information, and accurate molecular weight (mass deviation < 10 ppm) of the detected signals with the corresponding information in the MWDB. Before formal identification, the chromatographic peaks of each lipid in different samples were subjected to integral correction to ensure the consistency of RT and ion flow intensity. Quality control (QC) samples were used to verify the stability of the identification system during the detection process. The optimized MRM transition parameters, including declustering potential (DP) and collision energy (CE), for each lipid were used to improve the specificity and accuracy of identification.

The data was subjected to unit variance scaling before performing unsupervised principal component analysis (PCA) using the statistics function prcomp in R version 3.5.1 (www.r-project.org, accessed on 26 May 2026). Pearson correlation coefficients (PCCs) between samples were calculated using the corfunction in R and presented only as heatmaps. The hierarchical cluster analysis (HCA) results of samples and metabolites were presented as heatmaps with dendrograms. Both HCA and PCC were carried out by R package pheatmap. Orthogonal partial least squares discriminant analysis (OPLS-DA) was performed using R package MetaboAnalystR. The robustness of the OPLS-DA model was validated by 200 permutation tests. Differential lipids were screened with the criteria of variable importance in projection (VIP) > 1 from the OPLS-DA model and a Student’s *t*-test *p* value < 0.05. Screening criteria: *p* < 0.01, metabolites with fold-change (FC) ≥ 2 and ≤0.5. The KEGG Pathway database (http://www.kegg.jp/kegg/pathway.html, accessed on 26 May 2026) was used to annotate the differential metabolites. All relevant original data have been included as Supplementary Materials (Supplementary Material S1).

### 2.9. Statistics

Meat quality, chemical and mineral composition, fatty acids, amino acids, electronic nose and electronic tongue were assessed by one-way ANOVA using SPSS 26.0. When effects were significant (*p* < 0.05), Tukey’s multiple range test was used to identify significant differences among the categories. The tabulated results are shown as means ± standard error of the mean (SEM). Seven main differential flavor compounds were identified based on relevant literature and our data results, with *p* < 0.05. Spearman’s rank correlation analysis was performed to determine the relationships between the meat quality, chemical and mineral composition, fatty acids, amino acids, electronic nose, electronic tongue and DELs. Clustering Spearman’s correlation heatmap with signs was performed using the Metware Cloud at https://cloud.metware.cn. All means and comparison groups were considered statistically significant at *p* < 0.05.

## 3. Results

### 3.1. Meat Quality in LT of Mongolian Horses

Table 1 showed that Wushen Horses had the highest pH_45min_ and the lowest cooking loss compared with the other categories (*p* < 0.01). Barhu Horses had the highest b*_45min_ compared with the other categories (*p* < 0.01). Compared with the Baicha Horses and Ujimqin Horses, Wushen Horses induced a lower L*_45min_ (*p* < 0.05). Moreover, compared with the Barhu Horses, Wushen Horses induced a higher WHC (*p* < 0.05).

### 3.2. Chemical and Mineral Composition in LT of Mongolian Horses

Table 2 showed that the most outstanding characteristics of the chemical composition of the LT are the low fat (1.89–2.40%) and high protein contents (22.47–22.91%). It was also observed that among the four Mongolian horse categories, the most abundant macro-element detected was P, and the most abundant micro-element was Fe. Compared with Barhu Horses and Baicha Horses, Wushen Horses and Ujimqin Horses increased the fat and Ca contents of the LT (*p* < 0.01), Wushen increased the Fe content (*p* < 0.01) and decreased the moisture content (*p* < 0.05) of the LT. Moreover, compared with Baicha Horses, Wushen Horses and Ujimqin Horses increased the Cu contents of the LT (*p* < 0.01).

### 3.3. Fatty Acids Composition in LT of Mongolian Horses

Table 3 showed that Baicha Horses had the highest C12:0 compared with the other categories, and Barhu Horses had the highest C20:2 compared with the other categories (*p* < 0.01). Compared with Barhu Horses, Baicha Horses increased the C18:1n9c of the LT (*p* < 0.05), Wushen Horses and Ujimqin Horses decreased the C20:1 of the LT (*p* < 0.01). Compared with Wushen Horses and Barhu Horses, Ujimqin Horses decreased the C23:0 of the LT (*p* < 0.05). It was also observed that three the most abundant fatty acids detected in the four Mongolian horse categories were C16:0, C18:1n9c and C18:2n6c. Among SFA, the most abundant ones were C16:0 and C18:0, accounting for more than 80% of the total SFA content. Among MUFA, the most abundant ones were C18:1n9c. Among PUFA, the most abundant ones were C18:2n6c and C18:3n3. The n-6/n-3 PUFA ratio was 1.86–2.03, which was within the reasonable range of 1.5–2.0.

### 3.4. Amino Acid Composition in LT of Mongolian Horses

Table 4 showed that Wushen Horses had the highest serine, glutamic acid, ∑FAA, ∑NEAA, ∑TAA and NEAA/TAA, and had the lowest EAA/TAA and EAA/NEAA compared with the other categories (*p* < 0.01). Compared with Wushen Horses, Barhu Horses increased the methionine and decreased the aspartic acid and glycine of the LT (*p* < 0.05), Barhu Horses and Ujimqin Horses decreased the arginine of the LT (*p* < 0.05), Baicha Horses and Ujimqin Horses decreased the tyrosine (*p* < 0.05) and ∑SAA (*p* < 0.01) of the LT. Compared with Barhu Horses, Ujimqin Horses decreased the proline of the LT (*p* < 0.05) and Baicha Horses decreased the ∑BAA of the LT (*p* < 0.05). It was also observed that the two most abundant essential amino acids (EAA) detected in the four Mongolian horse categories were leucine and lysine, while the two most abundant non-essential amino acids (NEAA) were aspartic acid and glutamic acid.

### 3.5. Electronic Nose and Electronic Tongue Analysis in LT of Mongolian Horses

**Figure 1:**
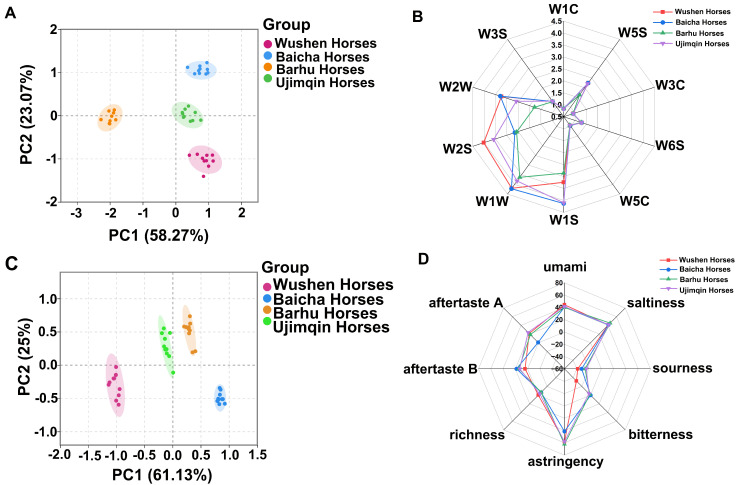
Differences in odor profile and taste profile among the four Mongolian horse categories. (**A**) Electronic nose PCA diagram; (**B**) radar diagram of odor response values; (**C**) electronic tongue PCA diagram; (**D**) radar diagram of taste values. n = 10.

Figure 1A shows the results of the PCA based on electronic nose response values of the LT from four Mongolian horse categories. The cumulative contribution rate of the first two principal components (PC1 = 58.27%, PC2 = 23.07%) reached 81.34%, indicating that they could effectively characterize the differences in flavor characteristics among samples. Although the confidence ellipse of the LT between Barhu Horses and Ujimqin Horses showed partial overlap, they were still different. The flavor characteristics of the LT of Wushen Horses and Baicha Horses could be clearly separated in the plot. The radar plot of Figure 1B and Table S1 further showed the differences in the response values of the electronic nose of the LT from the four Mongolian horse categories We detected large differences among these four categories in the responses of the W5S, W1S, W1W, W2S, and W2W sensors (*p* < 0.01). This indicates that the odor substances in the LT of the four Mongolian horse categories mainly include nitrogen oxides, methane, hydrocarbons, terpenes, sulfide compounds, alcohols, aldehydes, ketones, organic sulfides and aromatic compounds. In particular, compared with the other categories, the odor substances of Wushen Horses showed the highest response values to W2S sensors, while the odor substances of Barhu Horses showed the lowest response values to W5S, W1S, and W2W sensors (*p* < 0.01). Compared with the Wushen Horses and Baicha Horses, the odor substances of Barhu Horses decreased the response value to W1W sensors (*p* < 0.01).

As shown in the PCA of Figure 1C, the cumulative contribution rate of the first two principal components (PC1 = 61.13%, PC2 = 25%) reached 86.13%, indicating that there were certain differences in the taste of the LT among the four Mongolian horse categories. The radar plot (Figure 1D) and Table S2 further showed the differences in the response values of electronic tongue of the LT from the four Mongolian horse categories for saltiness, sourness, astringency, aftertaste astringency (aftertaste A), bitterness, aftertaste bitterness (aftertaste B), umami, and richness. Among them, the astringency, saltiness, and umami response values were relatively high, indicating that the LT of the four Mongolian horse categories were rich in astringency, saltiness, and umami. The results of electronic tongue analysis showed that Wushen Horses had the highest umami and richness response values, and had the lowest sourness, bitterness and aftertaste B response values compared with the other categories (*p* < 0.01). Barhu Horses had the highest saltiness response values compared with the other categories (*p* < 0.01). Baicha Horses had the lowest astringency and aftertaste A response values compared with the other categories (*p* < 0.01).

### 3.6. Lipidomic Analysis

**Figure 2:**
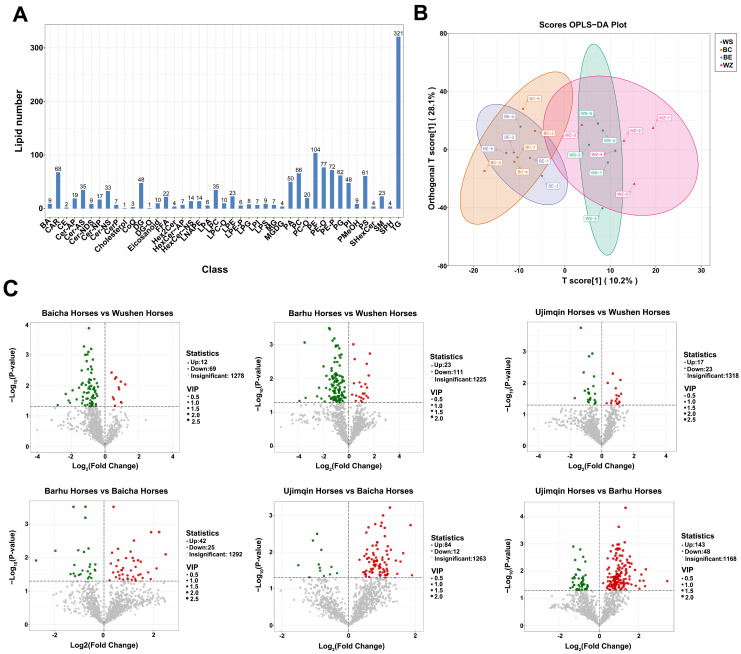
(**A**) Summary diagram of lipids; (**B**) OPLS-DA score of lipids; (**C**) volcano plot of differential lipids. n = 5.

Based on lipidomic analysis, a total of 1359 lipids were identified, categorized into 43 subgroups. The predominant lipid classes included triglycerides (TGs, 321 types), phosphatidylethanolamines (PEs, 253 types), and ceramides (CERs, 120 types), which collectively made up over 50% of the total lipid numbers (Figure 2A). We observed a clear separation from the OPLS-DA score plots among the four Mongolian horse categories. A permutation test (200 repetitions) confirmed the model’s robustness and reliability, yielding R^2^Y = 0.98, Q^2^ = 0.71. This analysis confirmed significant differences in the lipid profiles among the four Mongolian horse categories (Figure 2B). Based on screening criteria of VIP > 1 and *p* < 0.05, and FC > 2 or FC < 0.5, 163 DELs in the LT of the four Mongolian horse categories were identified. Among the 163 DELs, 25 were lysophosphatidylcholines (LPCs), 13 were lysophosphatidylethanolamines (LPEs), 12 were cylcarnitines (CARs), and 9 were phosphatidylglycerols (PGs) (Supplementary Material S2). In total, 81 DELs were identified between Wushen Horses and Baicha Horses, including 12 up-regulated and 69 down-regulated. A total of 67 DELs were identified between Barhu Horses and Baicha Horses, including 42 up-regulated and 25 down-regulated. Additionally, 134 DELs were identified between Barhu Horses and Wushen Horses, including 23 up-regulated and 111 down-regulated. Furthermore, 96 DELs were identified between Ujimqin Horses and Baicha Horses, including 84 up-regulated and 12 down-regulated. In total, 191 DELs were identified between Ujimqin Horses and Barhu Horses, including 143 up-regulated and 48 down-regulated. Forty DELs were identified between Wushen Horses and Ujimqin Horses, including 17 up-regulated and 23 down-regulated (Figure 2C). We found that the Wushen Horses had the highest and the Barhu Horses had the lowest levels of significantly DELs, such as LPC(18:2/0:0) (*p* < 0.01), LPC(0:0/18:2) (*p* < 0.01), LPC(22:5/0:0) (*p* < 0.05), and PC(16:0_16:0) (*p* < 0.05) (Table 5).

**Figure 3:**
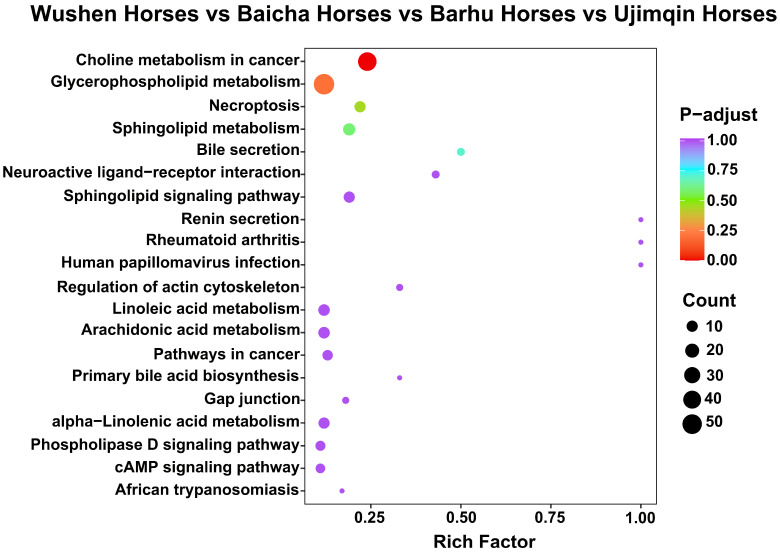
Differential lipid KEGG enrichment bubble plot. n = 5.

The KEGG enrichment analysis was employed to identify lipid metabolism pathways in the four Mongolian horse categories. As shown in Figure 3, this study analyzed 163 DELs. These DELs were significantly enriched in signaling pathways including glycerophospholipid metabolism, sphingolipid metabolism, bile secretion, neuroactive ligand-receptor interaction, sphingolipid signaling pathway, renin secretion, regulation of actin cytoskeleton, linoleic acid metabolism, arachidonic acid metabolism, pathways in cancer, primary bile acid biosynthesis, gap junction, alpha-linolenic acid metabolism, phospholipase D signaling pathway, and cAMP signaling pathway. Among these pathways, the metabolic pathways are specifically associated with lipid metabolism and thus deserve further in-depth analysis.

### 3.7. Correlation Analysis

**Figure 4:**
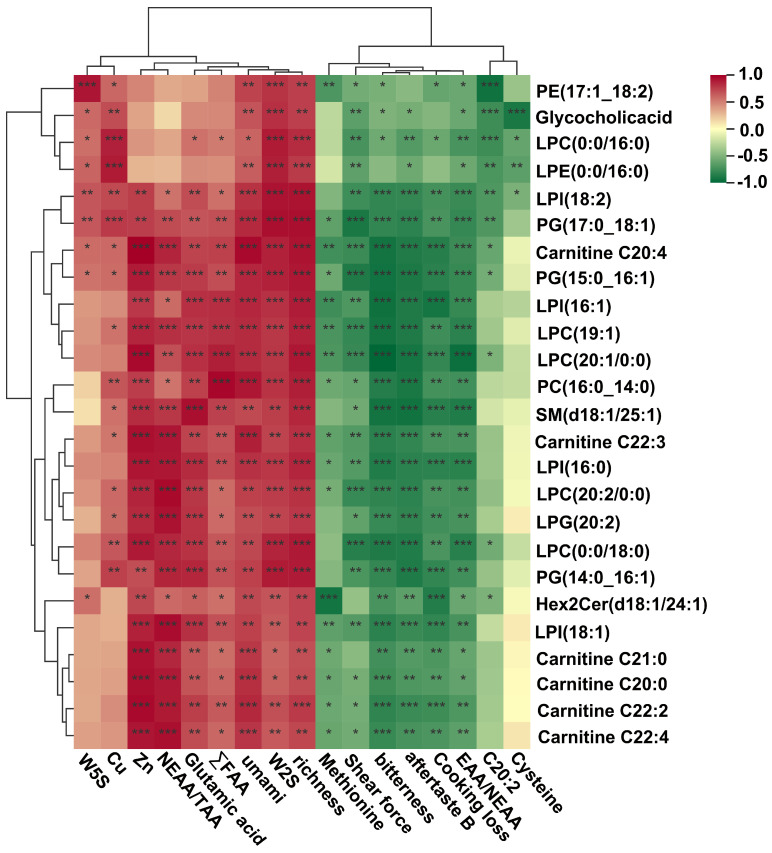
Correlation analyses. Asterisk (*) means *p*-values smaller than 0.05. Double asterisks (**) mean *p*-values smaller than 0.01. Asterisk (***) means *p*-values smaller than 0.001. n = 5.

To further explore the relationships between the significant DELs and the meat quality, chemical composition, fatty acids, amino acids, mineral contents, electronic nose, and electronic tongue traits, we performed a correlation analysis. Results with a correlation coefficient (r) larger than 0.4 or less than −0.4 were selected. The results showed that a total of 25 DELs were significantly associated with the aforementioned traits (|r| > 0.40, *p* < 0.05) (Figure 4, Supplementary Material S3). Specifically, all these DELs exhibited positive correlations with umami, W2S, and richness, while showing negative correlations with the EAA/NEAA. In addition, another 23 DELs were negatively correlated with the shear force and cooking loss (Figure 4). These DELs were enriched in metabolic pathways, glycerophospholipid metabolism, linoleic acid metabolism, arachidonic acid metabolism, and alpha-linolenic acid metabolism (Supplementary Material S2). Table 6 presented the correlation analysis results with |r| ≥ 0.85. Carnitine C20:4 was positively correlated with the Zn (r = 0.90; *p* < 0.001) and umami (r = 0.85; *p* < 0.001). LPC(20:1/0:0) was negatively correlated with bitterness (r = −0.90; *p* < 0.001) and EAA/NEAA (r = −0.86; *p* < 0.001), and was positively correlated with the Zn (r = 0.86; *p* < 0.001). PC(16:0_14:0) was positively correlated with the ∑FAA (r = 0.87; *p* < 0.001). Carnitine C22:2 was positively correlated with the Zn (r = 0.86; *p* < 0.001). LPC(20:2/0:0) was positively correlated with the NEAA/TAA (r = 0.86; *p* < 0.001). LPI(18:1) was positively correlated with the NEAA/TAA (r = 0.85; *p* < 0.001). Moreover, LPI(16:1) was negatively correlated with bitterness (r = −0.85; *p* < 0.001).

## 4. Discussion

Meat quality is often valued by parameters such as pH, color, cooking loss, tenderness, or TPA, which are key attributes influencing consumer preferences. The variation in muscle pH during slaughter is critical for meat preservation and processing, and also serve as one of the key indicators for assessing horse meat quality. Post-slaughter, the mechanism driving the decline in muscle pH involves the conversion of glycogen to lactic acid via the glycolytic pathway; subsequent accumulation of lactic acid within the muscle ultimately leads to a reduction in pH. Following the normal slaughter of livestock, the muscle pH is neutral or slightly alkaline. In this study, the pH of the LT from four categories of Mongolian horses all ranged from 5.60 to 5.90, falling within the reasonable variation range. Tateo et al. [25] reported that the pH_24h_ of horse meat decreased to 5.40–5.90, which was consistent with the findings of this study. Meat color is a crucial indicator of product freshness and plays a vital role in consumers’ perception of meat quality and their purchasing decisions. We found that the L*, a*, and b* values of the LT in the four Mongolian horse categories ranged from 26.42 to 29.94, 15.56 to 17.30, and 3.09 to 5.24, respectively, which were consistent with the previous study [26]. Among the categories, Wushen Horses had the highest pH and the lowest L* and b* values. The WHC, drip loss and cooking loss, reflects meat juiciness; notably, drip loss is a critical indicator of muscle water retention [27]. Barhu Horses exhibited significantly lower WHC and higher drip loss and cooking loss, indicating poorer water retention and juiciness. Tenderness, a key eating quality trait, is quantified by shear force, where lower values correspond to greater tenderness [25]. Chewiness and gumminess are negatively correlated with tenderness. In this study, Wushen Horses had the lowest shear force, chewiness, and gumminess, indicating superior tenderness, texture, and palatability. Collectively, these results suggest that among the four Mongolian horse categories investigated, Wushen Horses exhibit the best meat quality.

In comparison with other conventional types of meat, such as pork, beef, or mutton, horse meat exhibits excellent nutritional properties, specifically including a high content of protein (ranging from 21.16% to 22.44%) and water (ranging from 73.05% to 75.51%) and a low amount of fat (ranging from 0.86% to 2.0%) [6]. The moisture content in meat exerts a significant impact on the storage, processing, and quality characteristics (such as color, aroma, and taste) of meat products. Studies had shown that horse meat has a moisture content ranging from 74.78% in the meat of “Galician Mountain” foals slaughtered at 15 months to 77.40% in “Galician Mountain” foals slaughtered at 9 months of age from an extensive production system [27,28]. In this study, the moisture content of the LT in the four Mongolian horse categories ranged from 70.88 ± 1.15% to 72.48 ± 1.04%, which was lower to those reported. These differences in moisture content could be due to factors such as origin, breed, husbandry system, and age at slaughter. Among the components of fresh meat, the protein content is second only to moisture, accounting for approximately 20%. In addition, fat content is also a key factor affecting meat tenderness and texture. Within a certain range, a higher fat content results in tenderer meat with a better texture. Moreover, fat generates various aromatic compounds during cooking, which impart flavor to meat products [29]. Franco et al. [28] reported that the protein content in the LT of fresh horse meat ranged from 19.90% to 20.59% and the fat content of fresh horse meat was 2.56%, while Sarriés and Beriain [30] reported that the ash content in the LT of fresh horse meat ranged from 2.56% to 4.03%. These results were basically consistent with the findings of this study.

Fatty acids, composed of SFA, MUFA, and PUFA, are key indicators for measuring the sensory and nutritional characteristics of meat, and also exert an impact on consumers’ health. The content of SFA, MUFA and PUFA in meat exerts a positive effect on meat quality by significantly enhancing sensory attributes such as aroma, tenderness, and flavor, affects human cholesterol levels, and helps prevent cardiovascular diseases [31]. Tateo et al. [25] reported that the LT of adult horses contained approximately 44% SFA, 33% MUFA, and 23% PUFA, which was consistent with the findings of our study. The inherent properties of different fatty acids directly influence the color, flavor, and texture of horse meat. Our study found that C18:1n9c, C18:2n6c, and C18:3n3 were the dominant fatty acids in the LT across the four Mongolian horse categories. C18:1n9c exhibits high oxidative stability, which minimizes the formation of off-flavor compounds during cooking. Additionally, it can improve meat tenderness and juiciness [22]. From a health perspective, C18:1n9c is recognized as a beneficial fatty acid, as it can regulate serum cholesterol levels in humans, reduce the accumulation of low-density lipoprotein cholesterol, and lower the risk of cardiovascular diseases [32]. C18:2n6c and C18:3n3 are essential fatty acids for humans, as the human body cannot synthesize these compounds endogenously and thus must be obtained through dietary intake. During thermal processing, C18:2n6c and C18:3n3 participate in the Maillard reaction, generating aromatic compounds such as aldehydes and ketones that impart unique roasted or stewed flavors to Mongolian horse meat [33]. Compared with other meat types, horse meat contains higher levels of C18:2n6c and C18:3n3, which better meet human nutritional requirements and contribute to enhanced immune regulatory function [32]. Researchers have also focused extensively on the dietary n-6/n-3 PUFA ratio, as an imbalance in this ratio promotes the development of cardiovascular diseases, cancer, inflammation, and various autoimmune disorders [34]. It has long been established that dietary n-3 PUFA has influenced physiological processes in the human body. Our results showed that there were no significant differences in n-3 PUFA content among the LT of the four Mongolian horse categories. Furthermore, the n-6/n-3 PUFA ratio in the LT of the four Mongolian horse categories ranged from 1.86 to 2.03 in this study, which was below the upper limit recommended by the World Health Organization. Meanwhile, the PUFA/SFA ratio exceeded the 0.4 threshold considered a healthy standard by the UK Department of Health [35]. These proportional characteristics were largely consistent with the findings of the study by De et al. [36]. Importantly, this proportional advantage is significantly superior to that of traditional meats such as beef, highlighting the potential of Mongolian horse meat as a “functional meat” and providing a high-quality source of protein to optimize consumers’ dietary structures and reduce the risk of chronic diseases. In conclusion, the results of this study confirm that horse meat possesses a healthier fatty acid profile, characterized by balanced proportions of beneficial fatty acids and its compliance with key nutritional guidelines. This profile underscores the value of Mongolian horse meat as a nutritious and functionally relevant meat source.

Amino acids are the monomer unit in proteins synthesis and serve as essential prerequisites for vitamins and minerals to exert their full physiological functions. They are also key indicators for evaluating the physiological value of meat proteins, including attributes such as tenderness, juiciness, and flavor. EAAs, which cannot be synthesized endogenously by the human body and must be obtained through the diet, form the material basis for numerous critical physiological processes in humans [37]. The results of this study revealed the presence of 17 amino acids in the LT of the four Mongolian horse categories. These amino acids primarily included 9 EAAs (such as leucine, lysine, and arginine) and 8 NEAA (such as aspartic acid, glutamic acid, and alanine), and this pattern has also been observed in the study by Lorenzo et al. [6] on horse meat. Arginine was classified as a conditionally essential amino acid [37]. Amino acid proportional composition varies by meat species, and such variations not only shape meat protein nutritional value but also directly affect meat flavor. Generally, the closer the type, content, and proportion of EAAs in meat are to those in human proteins, the higher its nutritional value. Our results showed that the content and proportion of EAAs in the LT of the four Mongolian horse categories were well-balanced and aligned with the adult amino acid requirement standards proposed by the Food and Nutrition Board of the Institute of Medicine. Consequently, Mongolian horse meat can be regarded as an excellent source of high biological value proteins. When the ratio of EAA/NEAA falls within a reasonable range, it facilitates the absorption and utilization of amino acids. In our study, the EAA/NEAA ratio ranged from 1.12 to 1.19, which is consistent with the findings reported by Franco et al. [29]. The effects of amino acids on meat quality extend beyond contributing to meat flavor; but also improve meat quality by affecting muscle metabolism. Notably, glycine, alanine, serine, and threonine serve as key substrates for the sweet taste of meat, while aspartic acid and glutamic acid are essential for the umami taste of meat, both of which help enhance the sensory scores of meat products [38]. In horse meat, aspartic acid and glutamic acid are crucial for imparting umami. Specifically, glutamic acid can produce a “synergistic effect” with flavor nucleotides (such as inosine monophosphate and guanosine monophosphate), multiplying the intensity of umami and exerting a significant impact on the texture of meat. Studies by Gondret et al. [39] have shown that methionine can stimulate the synthesis and catabolism of proteins in muscle, and improve meat quality by enhancing antioxidant capacity. Wang et al. [40] reported that aspartic acid significantly increased the redness and decreased the shear force of the longissimus thoracis muscle in growing–finishing pigs. Additionally, Guo et al. [41] found that arginine increased the proportion of oxidative myofibers by activating SIRT1/AMPK pathway, and dietary supplementation of arginine and glutamic acid significantly improved the tenderness and juiciness of pork. In our study, the contents of arginine, aspartic acid, glutamic acid, glycine, serine, ∑FAA, and ∑SAA in Wushen Horses were higher than those in the other three Mongolian horse categories.

Mineral elements play a crucial role in biological processes such as metabolism, and oxidative stress responses in animals, and further influence sensory characteristics of meat, including flavor, tenderness, and color. Ca is a major component of animal bones and teeth, and it also participates in key physiological processes in animals, such as nerve signaling and muscle contraction. P is involved in biochemical processes like energy metabolism and DNA/RNA synthesis [42]. Cu is essential for the health of the human nervous system and blood vessels. Fe is responsible for the transport of oxygen and carbon dioxide in the human body, and it is vital for respiration and biological oxidation processes. Fe deficiency is the most widespread nutritional disorder worldwide, particularly in developing countries. Zn is a component of various enzymes in the human body, which participates in protein synthesis as well as the metabolism of carbohydrates and vitamin A, and offers significant benefits to human health [43]. Se is involved in the metabolism of thyroid hormones and immune regulation, and it also has the potential to prevent chronic diseases and cancer [44]. The results of this study indicated that the contents of Zn (80.68–93.38 mg/kg), P (669.06–685.12 mg/100 g), Fe (122.17–178.50 mg/kg), and Cu (3.61–5.06 mg/kg) in the LT of the four Mongolian horse categories were significantly higher than those in other meat types such as beef and pork [45]. Therefore, horse meat is considered an important source of minerals. Belhaj et al. [46] found that Se was the least abundant mineral component in meat, which was consistent with the results of this study. The sensory characteristics of meat are associated with the contents of minerals. Se, Zn and Cu have an anti-oxidation effect that can prevent lipid peroxidation and improve the quality of meat [47]. Wilborn et al. [48] reported that an increase in Ca content leads to a higher percentage of type I fibers and shifts muscle metabolism toward a more oxidative pathway, which decreases the L* and increases the a* of the LT of pigs. Our study found that the LT of Wushen Horses had the lowest L*, and this is speculated to be related to its Ca content.

The electronic tongue is an intelligent analytical technique that simulates the human gustatory perception system, enabling efficient detection of sourness, saltiness, bitterness and its aftertaste and astringency, as well as its umami and richness in samples. The electronic nose, an odor analysis instrument mimicking the olfactory system of mammals, can rapidly and non-destructively identify both simple and complex volatile compounds in samples. It has been widely applied in the freshness assessment and adulteration detection of different meat products, with the differences in its results being associated with variations in flavor substances [49]. Giovanelli et al. [50] demonstrated that the electronic nose could effectively distinguish three types of Italian dry-cured hams. In the present study, the application of electronic tongue and electronic nose techniques to detect the odor and taste characteristics of meat samples successfully achieved efficient differentiation among four horse meat samples. Furthermore, the results of this study showed that Wushen Horses exhibited a more abundant content of aromatic compounds and a higher umami response value, which might be related to their relatively high contents of LPCs and phosphatidylcholines (PCs). Previous studies have confirmed that PCs and LPCs are key lipids in the glycerophospholipid metabolic pathway and serve as the main sources of free fatty acids (FFAs) [51]. The degradation products of these phospholipids exert a significant impact on the flavor characteristics of meat. Specifically, LPCs are recognized as key precursors for various flavor compounds, and a relatively high LPC content can inhibit the volatilization of flavor substances and thereby preserve the flavor of meat [52].

Lipidomics, which employs high-throughput detection techniques such as mass spectrometry and chromatography, to systematically identify the species, structures, and contents of lipids in organisms. Lipids are one of the core indicators for meat quality evaluation, and the content and distribution of lipids in muscle directly affect key quality parameters of meat such as color, tenderness, juiciness, and flavor. GPs exert a significant influence on the flavor formation of meat [53]. Wang et al. [54] found that GPs had the highest content among lipids in the breast muscle of 43-week-old GuShi hens. Similarly, the present study also indicated that GPs were the most abundant lipid in the LT of Mongolian horses. Comparison of inter-group DELs showed: the most significant differences were between Wushen and Barhu Horses, and the smallest between Wushen and Ujimqin Horses. Additionally, Wushen and Ujimqin Horses had significantly higher contents of 3 key GPs (LPC(18:2/0:0), LPC(0:0/18:2), LPC(22:5/0:0)) than Barhu Horses. Xu et al. [53] confirmed that unsaturated fatty acids (UFAs) like 18:2 derived from LPCs are prone to oxidation into aldehydes and ketones during cooking, which provides lipid-level evidence for the superior meat quality of Wushen Horses. Notably, Barhu Horses had significantly lower TG(14:0_16:0_16:0) than Wushen Horses. Lower TGs, cause insufficient intramuscular fat (IMF). Since IMF correlates negatively with shear force, lower TGs tighten muscle fibers and increase chewing resistance [55], which explains the poorer tenderness of Barhu Horses. KEGG enrichment analysis identified glycerophospholipid metabolism, linoleic acid metabolism, arachidonic acid metabolism, and alpha-linolenic acid metabolism as core lipid metabolic pathways. Ma et al. [56] found that glycerophospholipid metabolism correlates closely with meat quality. As the primary source of FFAs in muscle, enriched glycerophospholipid metabolism indicates active phospholipid decomposition in Mongolian horse muscle. Lipids such as PEs and PCs in this pathway are key cell membrane components and release PUFAs via hydrolysis; oxidation products of PUFAs, such as 2,4-decadienal, are critical for the characteristic flavor of meat [57]. Enriched linoleic and alpha-linolenic acid metabolism reflects differential n-3 and n-6 PUFAs metabolic activity. Wushen Horses had significantly higher LPC(18:3) and LPC(20:3) than Barhu Horses. Higher C18:3 enhances muscle EPA and DHA synthesis, improving meat nutrition and tenderness by regulating membrane fluidity [58]. Enriched arachidonic acid metabolism suggests active prostaglandin synthesis, which reduces muscle fiber hardening via regulating cell apoptosis and inflammation, improving tenderness [59].

Correlation analysis revealed 25 DELs significantly associated with meat quality, amino acids, and minerals; some showed extremely high correlation strength, providing direct evidence to decipher the lipid regulatory mechanism for Mongolian horse meat quality. Specifically, carnitine C20:4 had a strong positive correlation with Zn content and umami. As a key lipase coenzyme, higher Zn promotes lipid hydrolysis. Meanwhile, Carnitine C20:4 enhances Zn bioavailability and generates umami-related aldehydes such as hexanal via oxidation, offering new clues for the “lipid-mineral-flavor” synergistic regulatory mechanism. LPC(20:1/0:0) showed strong negative correlations with bitterness and EAA/NEAA, plus a positive correlation with Zn. This indicates higher LPC(20:1/0:0) improves taste by inhibiting bitter substances and optimizes meat nutritional ratio via regulating amino acid metabolism balance. PC(16:0_14:0) was strongly positively correlated with total free amino acids (∑FAA), suggesting that phospholipid metabolism promotes the accumulation of umami amino acids such as glutamic acid and aspartic acid via amino acid synthesis precursors, thus enhancing meat umami. 23 DELs were negatively correlated with shear force and cooking loss, and lipids including LPE(18:2/0:0) and LPE(0:0/18:2) showed the most significant content differences.

## 5. Conclusions

In conclusion, meat quality and flavor differed among the four Mongolian horse categories, with Wushen Horses showing superior meat quality. All four groups exhibited rich fatty acids and amino acids profiles with balanced essential fatty acids and amino acids, indicating that these horses are high-quality protein sources. Specifically, Wushen Horses had higher pH_45min_, serine, glutamic acid, ∑FAA, ∑NEAA, ∑TAA, NEAA/TAA, W2S sensor response, umami, and richness values, as well as lower cooking loss, EAA/TAA, EAA/NEAA, sourness, bitterness, and aftertaste B values, reflecting better meat quality and flavor. In contrast, Barhu horses showed higher b*_45min_, C20:2, and saltiness values, and lower W5S, W1S, and W2W sensor responses. Lipidomics identified 163 differential lipids (DELs) as potential markers, including LPC(18:2/0:0) and PC(16:0_16:0). Correlation analysis indicated 23 DELs (e.g., carnitine C20:4) correlated positively with umami, W2S and richness, but negatively with shear force and cooking loss. Based on these findings, this study provides new insights into the molecular mechanisms underlying the meat quality and flavor of Mongolian horses.

## Figures and Tables

**Table 1:** Difference analysis of meat quality among the four Mongolian horse categories.

Items	Wushen Horses	Baicha Horses	Barhu Horses	Ujimqin Horses	*p*-Value
pH_45min_	5.89 ± 0.18 ^a^	5.65 ± 0.08 ^b^	5.64 ± 0.12 ^b^	5.65 ± 0.10 ^b^	<0.01
L*_45min_	26.42 ± 2.39 ^b^	29.94 ± 2.51 ^a^	28.42 ± 1.32 ^ab^	28.92 ± 1.10 ^a^	0.02
a*_45min_	17.30 ± 1.64	15.56 ± 1.39	15.68 ± 1.45	16.30 ± 1.53	0.05
b*_45min_	3.09 ± 0.76 ^b^	3.70 ± 1.04 ^b^	5.24 ± 0.72 ^a^	4.05 ± 1.02 ^b^	<0.01
Drip loss (%)	2.38 ± 0.36	2.73 ± 0.32	2.76 ± 0.43	2.60 ± 0.25	0.08
Cooking loss (%)	23.72 ± 1.50 ^b^	28.71 ± 2.44 ^a^	29.40 ± 2.92 ^a^	27.39 ± 3.06 ^a^	<0.01
Shear force (kgf)	6.70 ± 0.32	7.97 ± 0.69	7.94 ± 0.89	7.55 ± 0.89	0.22
WHC (%)	36.68 ± 1.30 ^a^	35.56 ± 2.21 ^ab^	33.81 ± 1.59 ^b^	35.99 ± 2.63 ^ab^	0.03
Cohesiveness (R, %)	0.32 ± 0.03	0.31 ± 0.03	0.29 ± 0.02	0.29 ± 0.02	0.06
Springiness (mm, %)	1.56 ± 0.08	1.70 ± 0.13	1.61 ± 0.20	1.57 ± 0.24	0.32
Gumminess (N, g)	18.80 ± 0.75	19.75 ± 0.95	21.66 ± 1.20	21.29 ± 0.85	0.42
Chewiness (mj, g)	29.43 ± 2.53	33.75 ± 2.48	32.28 ± 2.42	31.70 ± 2.87	0.33

^a,b^ Values within a row with different superscripts differ significantly at *p* < 0.05. Results were presented as mean ± SEM. n = 10.

**Table 2:** Difference analysis of chemical and mineral composition among the four Mongolian horse categories.

Items	Wushen Horses	Baicha Horses	Barhu Horses	Ujimqin Horses	*p*-Value
Chemical composition					
Moisture (%)	70.58 ± 0.97 ^b^	72.38 ± 1.64 ^a^	72.48 ± 1.04 ^a^	71.32 ± 1.22 ^ab^	0.03
Protein (%)	22.47 ± 1.25	22.57 ± 1.27	22.58 ± 0.87	22.91 ± 1.05	0.82
Fat (%)	2.40 ± 0.13 ^a^	1.95 ± 0.20 ^b^	1.89 ± 0.18 ^b^	2.21 ± 0.22 ^a^	<0.01
Ash (%)	1.95 ± 0.27	2.08 ± 0.25	2.13 ± 0.24	1.88 ± 0.24	0.12
Mineral composition (DM)					
Ca (mg/100 g)	38.34 ± 2.28 ^a^	32.61 ± 1.49 ^b^	33.97 ± 1.56 ^b^	36.77 ± 1.48 ^a^	<0.01
P (mg/100 g)	677.12 ± 11.03	685.12 ± 10.68	670.40 ± 12.24	669.06 ± 11.75	0.37
Cu (mg/kg)	4.90 ± 0.66 ^ab^	3.61 ± 0.46 ^c^	4.13 ± 0.67 ^bc^	5.06 ± 0.46 ^a^	<0.01
Fe (mg/kg)	148.50 ± 11.69 ^a^	127.17 ± 11.49 ^b^	134.50 ± 8.68 ^b^	139.00 ± 13.28 ^ab^	<0.01
Zn (mg/kg)	93.38 ± 12.98	82.98 ± 6.26	80.68 ± 10.27	80.73 ± 12.89	0.17
Se (mg/kg)	0.27 ± 0.04	0.25 ± 0.02	0.26 ± 0.03	0.28 ± 0.02	0.42

^a,b,c^ Values within a row with different superscripts differ significantly at *p* < 0.05. Results were presented as mean ± SEM. n = 10.

**Table 3:** Difference analysis of fatty acid composition among the four Mongolian horse categories (%).

Items	Wushen Horses	Baicha Horses	Barhu Horses	Ujimqin Horses	*p*-Value
C12:0	0.14 ± 0.02 ^b^	0.21 ± 0.04 ^a^	0.14 ± 0.03 ^b^	0.13 ± 0.03 ^b^	<0.01
C14:0	3.40 ± 0.89	2.84 ± 0.55	3.27 ± 0.47	3.40 ± 0.50	0.16
C15:0	0.20 ± 0.03	0.21 ± 0.04	0.23 ± 0.03	0.24 ± 0.04	0.09
C16:0	28.54 ± 3.85	30.40 ± 3.18	30.62 ± 2.96	29.90 ± 3.84	0.76
C17:0	0.39 ± 0.09	0.42 ± 0.09	0.44 ± 0.05	0.39 ± 0.08	0.50
C18:0	4.14 ± 0.86	4.00 ± 1.12	3.92 ± 0.82	3.98 ± 0.92	0.96
C20:0	0.10 ± 0.04	0.12 ± 0.04	0.11 ± 0.02	0.09 ± 0.02	0.12
C23:0	0.87 ± 0.20 ^a^	0.76 ± 0.13 ^ab^	0.90 ± 0.20 ^a^	0.67 ± 0.12 ^b^	0.01
C14:1	0.39 ± 0.07	0.32 ± 0.07	0.36 ± 0.11	0.35 ± 0.09	0.29
C16:1	5.86 ± 1.03	5.37 ± 0.94	5.88 ± 0.84	6.54 ± 1.10	0.09
C17:1	0.58 ± 0.05	0.58 ± 0.06	0.56 ± 0.05	0.61 ± 0.08	0.89
C18:1n9c	33.21 ± 2.51 ^ab^	35.39 ± 1.62 ^a^	30.58 ± 2.51 ^b^	33.91 ± 2.51 ^ab^	0.04
C20:1	0.32 ± 0.06 ^b^	0.37 ± 0.05 ^ab^	0.40 ± 0.06 ^a^	0.31 ± 0.05 ^b^	<0.01
C18:2n6c	10.81 ± 1.45	9.98 ± 2.07	9.95 ± 2.06	10.52 ± 1.31	0.64
C18:3n3	5.63 ± 0.56	5.51 ± 0.52	4.94 ± 0.64	5.42 ± 0.55	0.50
C20:2	0.09 ± 0.04 ^b^	0.12 ± 0.02 ^b^	0.18 ± 0.06 ^a^	0.09 ± 0.02 ^b^	<0.01
C20:3n6	0.25 ± 0.09	0.23 ± 0.07	0.27 ± 0.09	0.20 ± 0.05	0.24
C20:3n3	0.22 ± 0.07	0.17 ± 0.04	0.23 ± 0.08	0.22 ± 0.05	0.19
∑SFA ^1^	37.79 ± 2.99	38.95 ± 3.51	39.63 ± 3.51	38.78 ± 2.94	0.84
∑MUFA ^2^	40.36 ± 2.21	42.03 ± 2.58	37.76 ± 2.92	41.71 ± 2.67	0.09
∑PUFA ^3^	17.01 ± 2.56	15.44 ± 2.35	16.14 ± 2.92	16.44 ± 1.90	0.58
PUFA/SFA	0.45 ± 0.05	0.40 ± 0.05	0.41 ± 0.10	0.43 ± 0.05	0.30
∑n-3PUFA ^4^	5.86 ± 1.45	5.10 ± 1.54	5.73 ± 1.34	5.64 ± 0.90	0.46
∑n-6PUFA ^5^	11.15 ± 1.48	10.33 ± 2.07	10.40 ± 2.15	10.81 ± 1.33	0.72
n-6PUFA/n-3 PUFA	1.97 ± 0.36	2.03 ± 0.37	1.86 ± 0.41	1.95 ± 0.30	0.76

^1^ ∑SFA = ∑ (C12:0, C14:0, C15:0, C16:0, C17:0, C18:0, C20:0, C23:0). ^2^ ∑MUFA = ∑(C14:1, C16:1, C17:1, C18:1n9c, C20:1). ^3^ ∑PUFA = ∑(C18:2n6c, C18:3n3, C20:2, C20:3n6, C20:3n3). ^4^ n-6PUFA: total omega 6 fatty acids, ∑n-6PUFA = ∑(C18:2n6c, C20:2, C20:3n6). ^5^ n-3PUFA: total omega 3 fatty acids, ∑n-3PUFA = ∑(C18:3n3, C20:3n3). ^a,b^ Values within a row with different superscripts differ significantly at *p* < 0.05. Results were presented as mean ± SEM. n = 10.

**Table 4:** Difference analysis of amino acid composition among the four Mongolian horse categories (g/100 g DM).

Items	Wushen Horses	Baicha Horses	Barhu Horses	Ujimqin Horses	*p*-Value
Threonine	2.81 ± 0.12	2.77 ± 0.13	2.81 ± 0.29	2.73 ± 0.19	0.76
Valine	3.09 ± 0.14	3.15 ± 0.14	3.28 ± 0.22	3.15 ± 0.16	0.10
Methionine	1.54 ± 0.28 ^b^	1.62 ± 0.12 ^ab^	1.76 ± 0.07 ^a^	1.69 ± 0.10 ^ab^	0.03
Isoleucine	3.00 ± 0.12	2.92 ± 0.11	2.96 ± 0.28	2.89 ± 0.13	0.57
Leucine	5.71 ± 0.17	5.60 ± 0.27	5.80 ± 0.32	5.58 ± 0.25	0.23
Phenylalanine	3.30 ± 0.13	3.17 ± 0.12	3.35 ± 0.28	3.19 ± 0.16	0.12
Lysine	5.80 ± 0.19	5.72 ± 0.176	5.58 ± 0.37	5.63 ± 0.25	0.23
Histidine	2.78 ± 0.31	2.79 ± 0.10	2.98 ± 0.10	2.85 ± 0.18	0.10
Arginine	4.17 ± 0.14 ^a^	4.10 ± 0.15 ^ab^	3.96 ± 0.19 ^b^	3.96 ± 0.18 ^b^	0.01
Aspartic acid	5.94 ± 0.40 ^a^	5.58 ± 0.25 ^ab^	5.50 ± 0.51 ^b^	5.61 ± 0.21 ^ab^	0.04
Serine	2.24 ± 0.08 ^a^	1.93 ± 0.14 ^b^	2.02 ± 0.26 ^b^	2.04 ± 0.19 ^b^	<0.01
Glutamic acid	9.49 ± 0.20 ^a^	8.17 ± 0.30 ^b^	8.59 ± 0.57 ^b^	8.55 ± 0.29 ^b^	<0.01
Glycine	2.88 ± 0.24 ^a^	2.72 ± 0.13 ^ab^	2.64 ± 0.18 ^b^	2.72 ± 0.14 ^ab^	0.03
Alanine	4.23 ± 0.25	4.08 ± 0.24	4.34 ± 0.26	4.08 ± 0.30	0.09
Cysteine	1.16 ± 0.15	1.23 ± 0.10	1.24 ± 0.22	1.14 ± 0.09	0.31
Tyrosine	1.33 ± 0.15 ^a^	1.17 ± 0.10 ^b^	1.23 ± 0.15 ^ab^	1.14 ± 0.09 ^b^	0.01
Proline	1.87 ± 0.15 ^ab^	1.83 ± 0.11 ^ab^	2.00 ± 0.22 ^a^	1.75 ± 0.11 ^b^	0.01
∑FAA ^1^	15.44 ± 0.44 ^a^	13.75 ± 0.52 ^b^	14.08 ± 0.85 ^b^	14.15 ± 0.39 ^b^	<0.01
∑SAA ^2^	16.32 ± 0.52 ^a^	15.60 ± 0.45 ^b^	15.76 ± 0.74 ^ab^	15.53 ± 0.57 ^b^	0.02
∑BAA ^3^	19.42 ± 0.80 ^ab^	19.25 ± 0.26 ^b^	20.12 ± 0.96 ^a^	19.36 ± 0.66 ^ab^	0.04
∑EAA ^4^	32.20 ± 1.11	31.84 ± 1.45	32.47 ± 1.47	31.68 ± 1.13	0.39
∑NEAA ^5^	29.14 ± 0.79 ^a^	26.71 ± 0.90 ^b^	27.52 ± 1.07 ^b^	27.02 ± 0.64 ^b^	<0.01
∑TAA ^6^	61.34 ± 1.79 ^a^	58.55 ± 1.24 ^b^	59.99 ± 2.31 ^b^	58.70 ± 1.37 ^b^	<0.01
EAA/TAA	0.52 ± 0.01 ^b^	0.54 ± 0.01 ^a^	0.54 ± 0.01 ^a^	0.54 ± 0.01 ^a^	<0.01
NEAA/TAA	0.48 ± 0.01 ^a^	0.46 ± 0.01 ^b^	0.46 ± 0.01 ^b^	0.46 ± 0.01 ^b^	<0.01
EAA/NEAA	1.10 ± 0.02 ^b^	1.19 ± 0.03 ^a^	1.18 ± 0.04 ^a^	1.17 ± 0.05 ^a^	<0.01

^1^ FAA: fresh amino acids, ∑FAA = ∑(aspartic acid, glutamic acid). ^2^ SAA: sweet amino acids, ∑SAA = ∑ (glycine, alanine, serine, arginine, threonine). ^3^ BAA: bitter amino acids, ∑BAA = ∑ (valine, methionine, isoleucine, leucine, phenylalanine, histidine). ^4^ EAA: essential amino acids, ∑EAA = ∑ (threonine, valine, methionine, isoleucine, leucine, phenylalanine, lysine, histidine). ^5^ NEAA: non-essential amino acids, ∑NEAA = ∑(aspartic acid, serine, glutamic acid, glycine, alanine, cysteine, tyrosine, proline, arginine). ^6^ TAA: total amino acids, ∑TAA =∑EAA + ∑NEAA. ^a,b^ Values within a row with different superscripts differ significantly at *p* < 0.05. Results were presented as mean ± SEM. n = 10.

**Table 5:** Significant DELs of LT in the four Mongolian horse categories (top 20).

Items	Wushen Horses	Baicha Horses	Barhu Horses	Ujimqin Horses	*p*-Value
LPC(18:3)	8373.34 ± 1590.07 ^a^	3725.61 ± 1167.06 ^b^	5396.63 ± 3106.52 ^ab^	8391.72 ± 2301.85 ^a^	<0.01
LPC(20:3)	4129.74 ± 1526.41 ^a^	2516.62 ± 1085.16 ^ab^	1796.92 ± 577.36 ^b^	3576.69 ± 542.66 ^a^	0.01
LPC(18:2/0:0)	4734.80 ± 2213.01 ^a^	2457.85 ± 672.84 ^ab^	1781.64 ± 351.71 ^b^	4633.48 ± 1500.57 ^a^	<0.01
LPC(0:0/18:2)	4774.84 ± 2194.93 ^a^	2467.75 ± 634.93 ^ab^	1801.61 ± 332.28 ^b^	4601.22 ± 1412.01 ^a^	<0.01
LPC(22:5/0:0)	2557.18 ± 948.57 ^a^	1527.55 ± 477.87 ^ab^	1087.09 ± 279.55 ^b^	1941.51 ± 513.49 ^ab^	0.01
LPC(0:0/22:5)	2477.38 ± 827.16 ^a^	1484.58 ± 505.23 ^b^	1064.37 ± 282.31 ^b^	1938.54 ± 469.24 ^ab^	<0.01
LPC(0:0/20:2)	844.14 ± 339.46 ^a^	459.87 ± 155.19 ^b^	296.98 ± 75.26 ^b^	644.48 ± 190.74 ^ab^	0.02
PC(16:0_16:0)	561.88 ± 152.38 ^a^	284.56 ± 111.16 ^b^	257.46 ± 72.92 ^b^	466.59 ± 237.40 ^ab^	0.03
TG(14:0_16:0_16:0)	280.67 ± 108.66 ^a^	169.31 ± 34.10 ^b^	180.56 ± 24.76 ^ab^	244.33 ± 37.95 ^ab^	<0.01
FFA(10:0)	360.75 ± 91.49 ^a^	315.08 ± 70.04 ^ab^	214.98 ± 22.67 ^b^	401.28 ± 72.99 ^a^	<0.01
LPC(20:4)	193.09 ± 43.84 ^a^	117.20 ± 40.74 ^bc^	88.03 ± 20.88 ^c^	159.40 ± 27.86 ^ab^	<0.01
PE(O-16:1_18:1)	82.46 ± 25.35 ^b^	93.09 ± 25.90 ^b^	184.32 ± 77.39 ^a^	76.11 ± 38.54 ^b^	0.01
LPE(0:0/18:2)	194.16 ± 51.84 ^a^	138.06 ± 22.25 ^ab^	113.94 ± 16.16 ^b^	195.80 ± 53.95 ^a^	0.01
LPE(18:2/0:0)	194.39 ± 52.81 ^a^	138.38 ± 22.48 ^ab^	113.54 ± 16.75 ^b^	194.71 ± 55.55 ^a^	0.02
LPC(0:0/22:4)	200.25 ± 69.40 ^a^	148.19 ± 52.54 ^ab^	93.89 ± 28.50 ^b^	170.35 ± 31.36 ^ab^	<0.01
PC(O-16:0_18:2)	87.83 ± 21.99 ^a^	67.73 ± 7.89 ^ab^	53.16 ± 7.19 ^b^	71.87 ± 12.22 ^ab^	<0.01
LPE(18:0/0:0)	76.98 ± 9.80 ^ab^	54.14 ± 13.84 ^b^	45.13 ± 18.47 ^b^	96.16 ± 35.21 ^a^	<0.01
FFA(17:1)	104.49 ± 40.48 ^a^	42.04 ± 11.92 ^b^	59.90 ± 19.24 ^b^	76.26 ± 15.78 ^ab^	<0.01
LPC(20:2/0:0)	62.13 ± 29.13 ^a^	19.39 ± 6.66 ^b^	21.23 ± 6.95 ^b^	31.05 ± 14.47 ^b^	0.04
MGDG(16:0_16:3)	95.12 ± 61.41 ^ab^	101.08 ± 42.48 ^ab^	39.65 ± 6.54 ^b^	162.92 ± 95.11 ^a^	<0.01

^a,b,c^ Values within a row with different superscripts differ significantly at *p* < 0.05. Results were presented as mean ± SEM. n = 10.

**Table 6:** Correlation analysis between differential lipids and meat quality, chemical components, fatty acids, amino acids, mineral contents, electronic nose as well as electronic tongue indices (|r| ≥ 0.85).

Differential Lipids	Meat Quality and Flavor Indicators	r-Value	*p*-Value
CarnitineC20:4	Zn	0.90	<0.001
LPC(20:1/0:0)	bitterness	−0.90	<0.001
PC(16:0_14:0)	∑FAA	0.87	<0.001
Carnitine C22:2	Zn	0.86	<0.001
LPC(20:2/0:0)	NEAA/TAA	0.86	<0.001
LPC(20:1/0:0)	Zn	0.86	<0.001
LPC(20:1/0:0)	EAA/NEAA	−0.86	<0.001
Carnitine C20:4	umami	0.85	<0.001
LPI(18:1)	NEAA/TAA	0.85	<0.001
LPI(16:1)	bitterness	−0.85	<0.001

## Data Availability

The original contributions presented in the study are included in the article/Supplementary Materials; further inquiries can be directed to the corresponding author.

## Supplementary Materials

The following supporting information can be downloaded at: https://www.mdpi.com/article/10.3390/foods15112044/s1, Supplementary Material S1: All relevant original data; Supplementary Material S2: Significantly DELs of MLD in the four Mongolian horse categories; Supplementary Material S3: Correlation analysis between DELs and meat quality, chemical components, fatty acids, amino acids, mineral contents, electronic nose as well as electronic tongue indices (|r| ≥ 0.40); Supplementary Tables: Table S1 Odour response values of LT in the four Mongolian horses categories, Table S2 Taste response values of LT in the four Mongolian horses categories.
