# Supplementary material for: Comparative Analysis of Meat Quality and Flavor Among Four Categories of Mongolian Horses
Source: Foods. 2026 Jun 5;15(11):2044. doi: 10.3390/foods15112044 (PMC13256809; doi:10.3390/foods15112044)
Supplement: Supplementary file 1 [file foods-15-02044-s001.zip › foods-4318301-Supplementary Materials/Supplementary Material S3.pdf]

Supplementary Material S3

Correlation analysis between DELs and meat quality, chemical components, fatty acids, amino acids, mineral contents, electronic nose as well as electronic tongue indices (|r| ≥ 0.40)

DELs	Meat quality and flavor indicators	r-value	*p*-value
PE(17:1_18:2)	W5S	0.82	<0.001
PE(17:1_18:2)	Cu	0.53	<0.05
PE(17:1_18:2)	umami	0.66	<0.01
PE(17:1_18:2)	W2S	0.72	<0.001
PE(17:1_18:2)	richness	0.67	<0.01
PE(17:1_18:2)	Methionine	-0.63	<0.01
PE(17:1_18:2)	Shear force	-0.50	<0.05
PE(17:1_18:2)	bitterness	-0.47	<0.05
PE(17:1_18:2)	Cooking loss	-0.54	<0.05
PE(17:1_18:2)	EAA/NEAA	-0.52	<0.05
PE(17:1_18:2)	C20:2	-0.84	<0.001
Glycocholicacid	W5S	0.53	<0.05
Glycocholicacid	Cu	0.63	<0.01
Glycocholicacid	umami	0.60	<0.01
Glycocholicacid	W2S	0.73	<0.001
Glycocholicacid	richness	0.70	<0.01
Glycocholicacid	Shear force	-0.58	<0.01
Glycocholicacid	bitterness	-0.46	<0.05
Glycocholicacid	aftertaste B	-0.48	<0.05
Glycocholicacid	EAA/NEAA	-0.53	<0.05
Glycocholicacid	C20:2	-0.68	<0.001
Glycocholicacid	Cysteine	-0.80	<0.001
LPC(0:0/16:0)	W5S	0.51	<0.05
LPC(0:0/16:0)	Cu	0.78	<0.001
LPC(0:0/16:0)	Glutamic acid	0.50	<0.05
LPC(0:0/16:0)	∑FAA	0.46	<0.05
LPC(0:0/16:0)	umami	0.54	<0.05
LPC(0:0/16:0)	W2S	0.80	<0.001
LPC(0:0/16:0)	richness	0.74	<0.001
LPC(0:0/16:0)	Shear force	-0.65	<0.01
LPC(0:0/16:0)	bitterness	-0.51	<0.05
LPC(0:0/16:0)	aftertaste B	-0.61	<0.01
LPC(0:0/16:0)	Cooking loss	-0.52	<0.05
LPC(0:0/16:0)	EAA/NEAA	-0.65	<0.01
LPC(0:0/16:0)	C20:2	-0.68	<0.001
LPC(0:0/16:0)	Cysteine	-0.55	<0.05
LPE(0:0/16:0)	W5S	0.54	<0.05
LPE(0:0/16:0)	Cu	0.80	<0.001
LPE(0:0/16:0)	umami	0.57	<0.01
LPE(0:0/16:0)	W2S	0.78	<0.001
LPE(0:0/16:0)	richness	0.62	<0.001

LPE(0:0/16:0)	Shear force	-0.57	<0.01
LPE(0:0/16:0)	aftertaste B	-0.52	<0.05
LPE(0:0/16:0)	EAA/NEAA	-0.52	<0.05
LPE(0:0/16:0)	C20:2	-0.63	<0.01
LPE(0:0/16:0)	Cysteine	-0.57	<0.01
LPI(18:2)	W5S	0.59	<0.01
LPI(18:2)	Cu	0.64	<0.01
LPI(18:2)	Zn	0.68	<0.01
LPI(18:2)	NEAA/TAA	0.49	<0.05
LPI(18:2)	Glutamic acid	0.63	<0.01
LPI(18:2)	∑FAA	0.52	<0.05
LPI(18:2)	umami	0.72	<0.001
LPI(18:2)	W2S	0.82	<0.001
LPI(18:2)	richness	0.82	<0.001
LPI(18:2)	Shear force	-0.68	<0.01
LPI(18:2)	bitterness	-0.72	<0.001
LPI(18:2)	aftertaste B	-0.72	<0.001
LPI(18:2)	Cooking loss	-0.66	<0.01
LPI(18:2)	EAA/NEAA	-0.70	<0.001
LPI(18:2)	C20:2	-0.62	<0.01
LPI(18:2)	Cysteine	-0.47	<0.05
PG(17:0_18:1)	W5S	0.59	<0.01
PG(17:0_18:1)	Cu	0.69	<0.001
PG(17:0_18:1)	Zn	0.68	<0.01
PG(17:0_18:1)	NEAA/TAA	0.63	<0.01
PG(17:0_18:1)	Glutamic acid	0.60	<0.01
PG(17:0_18:1)	∑FAA	0.61	<0.01
PG(17:0_18:1)	umami	0.75	<0.01
PG(17:0_18:1)	W2S	0.84	<0.001
PG(17:0_18:1)	richness	0.84	<0.001
PG(17:0_18:1)	Methionine	-0.56	<0.05
PG(17:0_18:1)	Shear force	-0.80	<0.001
PG(17:0_18:1)	bitterness	-0.72	<0.001
PG(17:0_18:1)	aftertaste B	-0.74	<0.001
PG(17:0_18:1)	Cooking loss	-0.65	<0.01
PG(17:0_18:1)	EAA/NEAA	-0.71	<0.001
PG(17:0_18:1)	C20:2	-0.62	<0.01
Carnitine C20:4	W5S	0.52	<0.05
Carnitine C20:4	Cu	0.53	<0.05
Carnitine C20:4	Zn	0.90	<0.001
Carnitine C20:4	NEAA/TAA	0.77	<0.001
Carnitine C20:4	Glutamic acid	0.65	<0.01
Carnitine C20:4	∑FAA	0.67	<0.01
Carnitine C20:4	umami	0.85	<0.001
Carnitine C20:4	W2S	0.77	<0.001
Carnitine C20:4	richness	0.83	<0.001
Carnitine C20:4	Methionine	-0.67	<0.01
Carnitine C20:4	Shear force	-0.69	<0.001
Carnitine C20:4	bitterness	-0.83	<0.001

Carnitine C20:4	aftertaste B	-0.79	<0.001
Carnitine C20:4	Cooking loss	-0.77	<0.001
Carnitine C20:4	EAA/NEAA	-0.69	<0.001
Carnitine C20:4	C20:2	-0.53	<0.05
PG(15:0_16:1)	W5S	0.49	<0.05
PG(15:0_16:1)	Cu	0.52	<0.05
PG(15:0_16:1)	Zn	0.80	<0.001
PG(15:0_16:1)	NEAA/TAA	0.70	<0.001
PG(15:0_16:1)	Glutamic acid	0.69	<0.001
PG(15:0_16:1)	∑FAA	0.62	<0.01
PG(15:0_16:1)	umami	0.76	<0.001
PG(15:0_16:1)	W2S	0.78	<0.001
PG(15:0_16:1)	richness	0.81	<0.001
PG(15:0_16:1)	Methionine	-0.51	<0.05
PG(15:0_16:1)	Shear force	-0.79	<0.001
PG(15:0_16:1)	bitterness	-0.84	<0.001
PG(15:0_16:1)	aftertaste B	-0.80	<0.001
PG(15:0_16:1)	Cooking loss	-0.75	<0.001
PG(15:0_16:1)	EAA/NEAA	-0.71	<0.001
PG(15:0_16:1)	C20:2	-0.49	<0.05
LPI(16:1)	Zn	0.70	<0.001
LPI(16:1)	NEAA/TAA	0.52	<0.05
LPI(16:1)	Glutamic acid	0.73	<0.001
LPI(16:1)	∑FAA	0.74	<0.001
LPI(16:1)	umami	0.76	<0.001
LPI(16:1)	W2S	0.72	<0.001
LPI(16:1)	richness	0.79	<0.001
LPI(16:1)	Methionine	-0.68	<0.01
LPI(16:1)	Shear force	-0.62	<0.01
LPI(16:1)	bitterness	-0.85	<0.001
LPI(16:1)	aftertaste B	-0.79	<0.001
LPI(16:1)	Cooking loss	-0.82	<0.001
LPI(16:1)	EAA/NEAA	-0.71	<0.001
LPC(19:1)	Cu	0.49	<0.05
LPC(19:1)	Zn	0.76	<0.001
LPC(19:1)	NEAA/TAA	0.72	<0.001
LPC(19:1)	Glutamic acid	0.71	<0.001
LPC(19:1)	∑FAA	0.72	<0.001
LPC(19:1)	umami	0.74	<0.001
LPC(19:1)	W2S	0.70	<0.001
LPC(19:1)	richness	0.78	<0.001
LPC(19:1)	Methionine	-0.64	<0.01
LPC(19:1)	Shear force	-0.71	<0.001
LPC(19:1)	bitterness	-0.83	<0.001
LPC(19:1)	aftertaste B	-0.79	<0.001
LPC(19:1)	Cooking loss	-0.68	<0.01
LPC(19:1)	EAA/NEAA	-0.74	<0.001
LPC(20:1/0:0)	Zn	0.86	<0.001
LPC(20:1/0:0)	NEAA/TAA	0.62	<0.01

LPC(20:1/0:0)	Glutamic acid	0.73	<0.001
LPC(20:1/0:0)	∑FAA	0.79	<0.001
LPC(20:1/0:0)	umami	0.75	<0.001
LPC(20:1/0:0)	W2S	0.70	<0.001
LPC(20:1/0:0)	richness	0.81	<0.001
LPC(20:1/0:0)	Methionine	-0.63	<0.01
LPC(20:1/0:0)	Shear force	-0.71	<0.001
LPC(20:1/0:0)	bitterness	-0.90	<0.001
LPC(20:1/0:0)	aftertaste B	-0.82	<0.001
LPC(20:1/0:0)	Cooking loss	-0.71	<0.001
LPC(20:1/0:0)	EAA/NEAA	-0.86	<0.001
LPC(20:1/0:0)	C20:2	-0.46	<0.05
PC(16:0_14:0)	Cu	0.59	<0.01
PC(16:0_14:0)	Zn	0.68	<0.001
PC(16:0_14:0)	NEAA/TAA	0.49	<0.05
PC(16:0_14:0)	Glutamic acid	0.67	<0.05
PC(16:0_14:0)	∑FAA	0.87	<0.001
PC(16:0_14:0)	umami	0.81	<0.001
PC(16:0_14:0)	W2S	0.71	<0.001
PC(16:0_14:0)	richness	0.79	<0.001
PC(16:0_14:0)	Methionine	-0.51	<0.05
PC(16:0_14:0)	Shear force	-0.54	<0.05
PC(16:0_14:0)	bitterness	-0.78	<0.001
PC(16:0_14:0)	aftertaste B	-0.81	<0.001
PC(16:0_14:0)	Cooking loss	-0.66	<0.01
PC(16:0_14:0)	EAA/NEAA	-0.67	<0.01
SM(d18:1/25:1)	Cu	0.50	<0.05
SM(d18:1/25:1)	Zn	0.74	<0.001
SM(d18:1/25:1)	NEAA/TAA	0.70	<0.001
SM(d18:1/25:1)	Glutamic acid	0.84	<0.001
SM(d18:1/25:1)	∑FAA	0.66	<0.01
SM(d18:1/25:1)	umami	0.64	<0.01
SM(d18:1/25:1)	W2S	0.68	<0.01
SM(d18:1/25:1)	richness	0.76	<0.001
SM(d18:1/25:1)	Shear force	-0.50	<0.05
SM(d18:1/25:1)	bitterness	-0.83	<0.001
SM(d18:1/25:1)	aftertaste B	-0.84	<0.001
SM(d18:1/25:1)	Cooking loss	-0.76	<0.001
SM(d18:1/25:1)	EAA/NEAA	-0.78	<0.001
Carnitine C22:3	Cu	0.48	<0.05
Carnitine C22:3	Zn	0.84	<0.001
Carnitine C22:3	NEAA/TAA	0.82	<0.001
Carnitine C22:3	Glutamic acid	0.66	<0.01
Carnitine C22:3	∑FAA	0.61	<0.01
Carnitine C22:3	umami	0.78	<0.001
Carnitine C22:3	W2S	0.66	<0.01
Carnitine C22:3	richness	0.73	<0.001
Carnitine C22:3	Methionine	-0.56	<0.05
Carnitine C22:3	Shear force	-0.61	<0.01

Carnitine C22:3	bitterness	-0.75	<0.001
Carnitine C22:3	aftertaste B	-0.72	<0.001
Carnitine C22:3	Cooking loss	-0.65	<0.01
Carnitine C22:3	EAA/NEAA	-0.65	<0.01
LPI(16:0)	Zn	0.82	<0.001
LPI(16:0)	NEAA/TAA	0.81	<0.001
LPI(16:0)	Glutamic acid	0.74	<0.001
LPI(16:0)	∑FAA	0.63	<0.01
LPI(16:0)	umami	0.72	<0.001
LPI(16:0)	W2S	0.70	<0.001
LPI(16:0)	richness	0.76	<0.001
LPI(16:0)	Methionine	-0.53	<0.05
LPI(16:0)	Shear force	-0.60	<0.01
LPI(16:0)	bitterness	-0.78	<0.001
LPI(16:0)	aftertaste B	-0.74	<0.001
LPI(16:0)	Cooking loss	-0.74	<0.001
LPI(16:0)	EAA/NEAA	-0.75	<0.001
LPC(20:2/0:0)	Cu	0.53	<0.05
LPC(20:2/0:0)	Zn	0.77	<0.001
LPC(20:2/0:0)	NEAA/TAA	0.86	<0.001
LPC(20:2/0:0)	Glutamic acid	0.70	<0.001
LPC(20:2/0:0)	∑FAA	0.52	<0.05
LPC(20:2/0:0)	umami	0.67	<0.01
LPC(20:2/0:0)	W2S	0.68	<0.001
LPC(20:2/0:0)	richness	0.72	<0.001
LPC(20:2/0:0)	Methionine	-0.48	<0.05
LPC(20:2/0:0)	Shear force	-0.71	<0.001
LPC(20:2/0:0)	bitterness	-0.74	<0.001
LPC(20:2/0:0)	aftertaste B	-0.74	<0.001
LPC(20:2/0:0)	Cooking loss	-0.65	<0.01
LPC(20:2/0:0)	EAA/NEAA	-0.67	<0.01
LPG(20:2)	Cu	0.52	<0.05
LPG(20:2)	Zn	0.75	<0.001
LPG(20:2)	NEAA/TAA	0.84	<0.001
LPG(20:2)	Glutamic acid	0.71	<0.001
LPG(20:2)	∑FAA	0.52	<0.05
LPG(20:2)	umami	0.63	<0.01
LPG(20:2)	W2S	0.65	<0.01
LPG(20:2)	richness	0.70	<0.001
LPG(20:2)	Shear force	-0.56	<0.05
LPG(20:2)	bitterness	-0.70	<0.001
LPG(20:2)	aftertaste B	-0.73	<0.001
LPG(20:2)	Cooking loss	-0.67	<0.01
LPG(20:2)	EAA/NEAA	-0.65	<0.01
LPC(0:0/18:0)	Cu	0.59	<0.01
LPC(0:0/18:0)	Zn	0.80	<0.001
LPC(0:0/18:0)	NEAA/TAA	0.76	<0.001
LPC(0:0/18:0)	Glutamic acid	0.72	<0.001
LPC(0:0/18:0)	∑FAA	0.59	<0.01

LPC(0:0/18:0)	umami	0.64	<0.01
LPC(0:0/18:0)	W2S	0.77	<0.001
LPC(0:0/18:0)	richness	0.80	<0.001
LPC(0:0/18:0)	Shear force	-0.76	<0.001
LPC(0:0/18:0)	bitterness	-0.79	<0.001
LPC(0:0/18:0)	aftertaste B	-0.78	<0.001
LPC(0:0/18:0)	Cooking loss	-0.64	<0.01
LPC(0:0/18:0)	EAA/NEAA	-0.76	<0.001
LPC(0:0/18:0)	C20:2	-0.50	<0.05
PG(14:0_16:1)	Cu	0.67	<0.01
PG(14:0_16:1)	Zn	0.62	<0.01
PG(14:0_16:1)	NEAA/TAA	0.77	<0.001
PG(14:0_16:1)	Glutamic acid	0.76	<0.001
PG(14:0_16:1)	∑FAA	0.61	<0.01
PG(14:0_16:1)	umami	0.67	<0.01
PG(14:0_16:1)	W2S	0.80	<0.001
PG(14:0_16:1)	richness	0.80	<0.001
PG(14:0_16:1)	Shear force	-0.63	<0.01
PG(14:0_16:1)	bitterness	-0.72	<0.001
PG(14:0_16:1)	aftertaste B	-0.79	<0.001
PG(14:0_16:1)	Cooking loss	-0.72	<0.001
PG(14:0_16:1)	EAA/NEAA	-0.67	<0.01
Hex2Cer(d18:1/24:1)	W5S	0.50	<0.05
Hex2Cer(d18:1/24:1)	Zn	0.64	<0.01
Hex2Cer(d18:1/24:1)	NEAA/TAA	0.53	<0.05
Hex2Cer(d18:1/24:1)	Glutamic acid	0.56	<0.05
Hex2Cer(d18:1/24:1)	∑FAA	0.50	<0.05
Hex2Cer(d18:1/24:1)	umami	0.64	<0.01
Hex2Cer(d18:1/24:1)	W2S	0.60	<0.01
Hex2Cer(d18:1/24:1)	richness	0.65	<0.01
Hex2Cer(d18:1/24:1)	Methionine	-0.83	<0.001
Hex2Cer(d18:1/24:1)	bitterness	-0.65	<0.01
Hex2Cer(d18:1/24:1)	aftertaste B	-0.57	<0.01
Hex2Cer(d18:1/24:1)	Cooking loss	-0.78	<0.001
Hex2Cer(d18:1/24:1)	EAA/NEAA	-0.53	<0.05
Hex2Cer(d18:1/24:1)	C20:2	-0.47	<0.05
LPI(18:1)	Zn	0.77	<0.001
LPI(18:1)	NEAA/TAA	0.85	<0.001
LPI(18:1)	Glutamic acid	0.72	<0.001
LPI(18:1)	∑FAA	0.61	<0.01
LPI(18:1)	umami	0.67	<0.01
LPI(18:1)	W2S	0.58	<0.01
LPI(18:1)	richness	0.65	<0.01
LPI(18:1)	Methionine	-0.57	<0.01
LPI(18:1)	Shear force	-0.60	<0.01
LPI(18:1)	bitterness	-0.75	<0.001
LPI(18:1)	aftertaste B	-0.70	<0.001
LPI(18:1)	Cooking loss	-0.72	<0.001
LPI(18:1)	EAA/NEAA	-0.65	<0.01

Carnitine C21:0	Zn	0.83	<0.001
Carnitine C21:0	NEAA/TAA	0.76	<0.001
Carnitine C21:0	Glutamic acid	0.60	<0.01
Carnitine C21:0	∑FAA	0.51	<0.05
Carnitine C21:0	umami	0.69	<0.001
Carnitine C21:0	W2S	0.55	<0.05
Carnitine C21:0	richness	0.62	<0.01
Carnitine C21:0	Methionine	-0.51	<0.05
Carnitine C21:0	bitterness	-0.67	<0.01
Carnitine C21:0	aftertaste B	-0.60	<0.01
Carnitine C21:0	Cooking loss	-0.63	<0.01
Carnitine C21:0	EAA/NEAA	-0.54	<0.05
Carnitine C20:0	Zn	0.84	<0.001
Carnitine C20:0	NEAA/TAA	0.80	<0.001
Carnitine C20:0	Glutamic acid	0.60	<0.01
Carnitine C20:0	∑FAA	0.54	<0.05
Carnitine C20:0	umami	0.73	<0.001
Carnitine C20:0	W2S	0.56	<0.05
Carnitine C20:0	richness	0.63	<0.01
Carnitine C20:0	Methionine	-0.53	<0.05
Carnitine C20:0	Shear force	-0.49	<0.05
Carnitine C20:0	bitterness	-0.68	<0.001
Carnitine C20:0	aftertaste B	-0.62	<0.01
Carnitine C20:0	Cooking loss	-0.61	<0.01
Carnitine C20:0	EAA/NEAA	-0.55	<0.05
Carnitine C22:2	Zn	0.86	<0.001
Carnitine C22:2	NEAA/TAA	0.81	<0.001
Carnitine C22:2	Glutamic acid	0.68	<0.01
Carnitine C22:2	∑FAA	0.58	<0.01
Carnitine C22:2	umami	0.75	<0.001
Carnitine C22:2	W2S	0.62	<0.01
Carnitine C22:2	richness	0.69	<0.001
Carnitine C22:2	Methionine	-0.52	<0.05
Carnitine C22:2	Shear force	-0.49	<0.05
Carnitine C22:2	bitterness	-0.74	<0.001
Carnitine C22:2	aftertaste B	-0.70	<0.001
Carnitine C22:2	Cooking loss	-0.68	<0.001
Carnitine C22:2	EAA/NEAA	-0.62	<0.01
Carnitine C22:4	Zn	0.82	<0.001
Carnitine C22:4	NEAA/TAA	0.84	<0.001
Carnitine C22:4	Glutamic acid	0.63	<0.01
Carnitine C22:4	∑FAA	0.53	<0.05
Carnitine C22:4	umami	0.71	<0.001
Carnitine C22:4	W2S	0.58	<0.01
Carnitine C22:4	richness	0.65	<0.01
Carnitine C22:4	Methionine	-0.53	<0.05
Carnitine C22:4	Shear force	-0.50	<0.05
Carnitine C22:4	bitterness	-0.70	<0.001
Carnitine C22:4	aftertaste B	-0.65	<0.01

Carnitine C22:4	Cooking loss	-0.63	<0.01
Carnitine C22:4	EAA/NEAA	-0.57	<0.01
