# Supplementary material for: Comparative Analysis of Meat Quality and Flavor Among Four Categories of Mongolian Horses
Source: Foods. 2026 Jun 5;15(11):2044. doi: 10.3390/foods15112044 (PMC13256809; doi:10.3390/foods15112044)
Supplement: Supplementary file 1 [file foods-15-02044-s001.zip › foods-4318301-Supplementary Materials/Supplementary Tables.pdf]

**Table S1**

Odour response values of LT in the four Mongolian horses categories

| Items | Wushen Horses | Baicha Horses | Barhu Horses | Ujimqin Horses | *p*-value |
|---|---|---|---|---|---|
| W1C | 0.84±0.02 | 0.84±0.01 | 0.84±0.03 | 0.83±0.01 | 0.48 |
| W5S | 2.25±0.10$^{a}$ | 2.24±0.13$^{a}$ | 1.63±0.04$^{b}$ | 2.21±0.08$^{a}$ | <0.01 |
| W3C | 0.92±0.01 | 0.92±0.02 | 0.91±0.01 | 0.92±0.03 | 0.17 |
| W6S | 1.29±0.04 | 1.29±0.03 | 1.27±0.03 | 1.29±0.02 | 0.61 |
| W5C | 0.96±0.03 | 0.96±0.01 | 0.94±0.02 | 0.95±0.01 | 0.30 |
| W1S | 3.24±0.06$^{b}$ | 4.13±0.08$^{a}$ | 2.86±0.10$^{c}$ | 4.10±0.07$^{a}$ | <0.01 |
| W1W | 4.20±0.27$^{a}$ | 4.22±0.25$^{a}$ | 3.63±0.32$^{b}$ | 3.82±0.33$^{ab}$ | <0.01 |
| W2S | 4.02±0.09$^{a}$ | 2.65±0.10$^{c}$ | 2.57±0.09$^{c}$ | 3.58±0.09$^{b}$ | <0.01 |
| W2W | 3.24±0.07$^{a}$ | 3.29±0.04$^{a}$ | 1.78±0.05$^{c}$ | 2.59±0.05$^{b}$ | <0.01 |
| W3S | 1.29±0.02 | 1.29±0.01 | 1.28±0.04 | 1.30±0.02 | 0.74 |

$^{a,b,c,d}$ Mean values within a row with no common superscript differ significantly ($P < 0.05$). Results were presented as mean ± SE. n = 10.

**Table S2**

Taste response values of LT in the four Mongolian horses categories

| Items | Wushen Horses | Baicha Horses | Barhu Horses | Ujimqin Horses | *p*-value |
|---|---|---|---|---|---|
| umami | 44.31±2.06[a] | 40.45±1.98[b] | 39.75±1.28[b] | 41.36±2.68[b] | <0.01 |
| saltiness | 41.15±0.80[b] | 41.31±1.51[b] | 45.23±1.57[a] | 41.70±1.97[b] | <0.01 |
| sourness | -38.36±5.31[c] | -32.02±3.90[b] | -26.51±4.32[a] | -24.46±3.59[a] | <0.01 |
| bitterness | -32.40±2.53[b] | 0.26±0.04[a] | -1.50±0.04[a] | -3.60±0.21[a] | <0.01 |
| astringency | 58.18±3.75[a] | 41.46±4.87[b] | 62.24±5.98[a] | 58.06±5.46[a] | <0.01 |
| richness | 0.21±0.05[a] | -6.23±0.18[c] | -6.32±0.14[c] | -3.10±0.20[b] | <0.01 |
| aftertaste B | 4.14±0.22[d] | 18.13±1.09[a] | 14.20±0.65[b] | 12.79±0.57[c] | <0.01 |
| aftertaste A | 21.19±1.77[b] | 0.70±0.11[d] | 18.46±0.98[c] | 23.76±1.25[a] | <0.01 |

[a,b,c,d] Mean values within a row with no common superscript differ significantly ($P < 0.05$). Results were presented as mean ± SE. n = 10.
